# Removal of infected pacemaker leads using an endoscopic minimally invasive cardiac surgical approach: a case report

**DOI:** 10.1186/s44215-024-00156-4

**Published:** 2024-06-24

**Authors:** Shuji Nagatomi, Takashi Oshitomi, Daiki Teduka, Shizuya Shintomi, Toshiharu Sasa, Hidetaka Murata, Kentaro Takaji, Ichiro Ideta, Hideyuki Uesugi

**Affiliations:** Department of Cardiovascular Surgery, Saiseikai Kumamoto Hospital, 5-3-1, Chikami, Minami-Ku, Kumamoto, 861-4193 Japan

**Keywords:** Minimally invasive cardiac surgery, Cardiac implantable electronic devices, Transvenous lead extraction, Surgical lead extraction

## Background

Cardiac implantable electronic device (CIED) leads occasionally need to be removed when infected or in the case of lead failure. Transvenous lead extraction (TLE) using an excimer laser or mechanical sheath is often performed; however, when that approach is difficult, surgical removal through a sternotomy is required. The risk of deep sternal wound infection (DSWI) is especially high in cases in which the infected site is close to the sternum. We report a case in which surgical removal of CIED leads via a thoracoscopic approach without a sternotomy was effective.

## Case presentation

The patient was a 52-year-old man with complete atrioventricular block. A skin fistula with purulent drainage developed above the pacemaker (PM) generator in the left anterior thoracic region and a device infection was suspected.

**Fig. 1:**
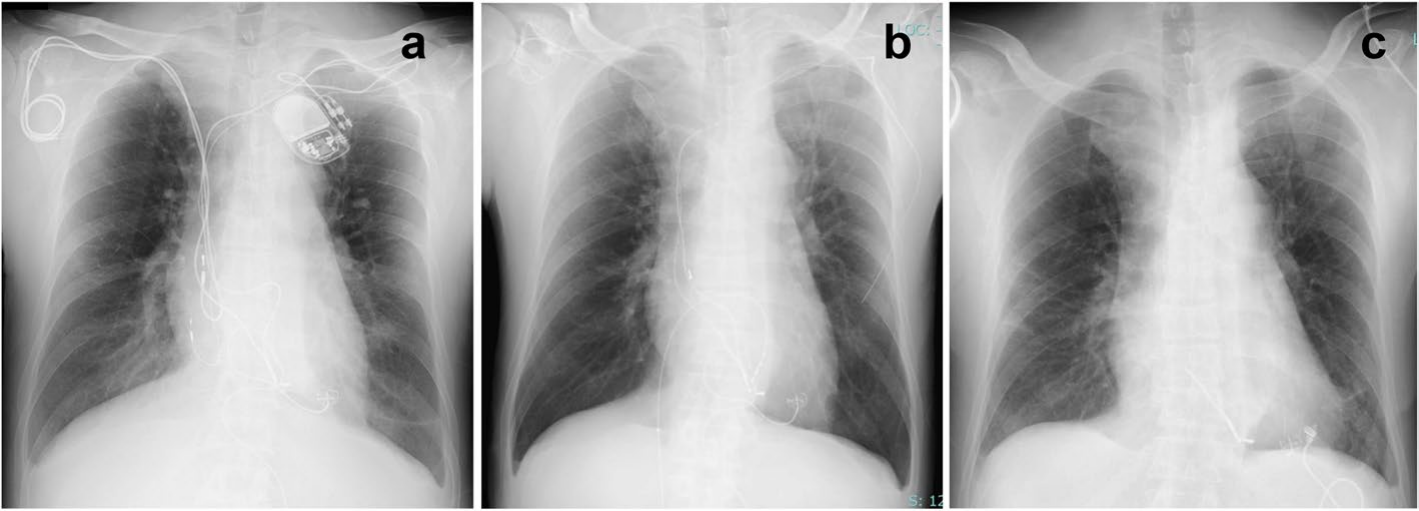
Perioperative chest radiography. A toral of four leads were placed transvenously and an epicardial lead was left in the diaphragmatic plane (**a**). Two RV leads was remained after transvenous lead extraction (**b**). Only a part of the RV lead remained and a new epicardial lead was placed (**c**)

He had a PM implanted via lower sternotomy and the generator was placed in his abdomen at the age of 11. Twenty-eight years ago (at age 23), a PM was reimplanted transvenously from the right anterior thoracic region. A PM reimplantation was made to the left anterior thoracic region due to skin thinning at age 39, and 2 leads was remained. Thus, a total of four transvenous leads were implanted (one right atrial (RA)/ right ventricular (RV) lead from each side) (Fig. 1a).

**Fig. 2:**
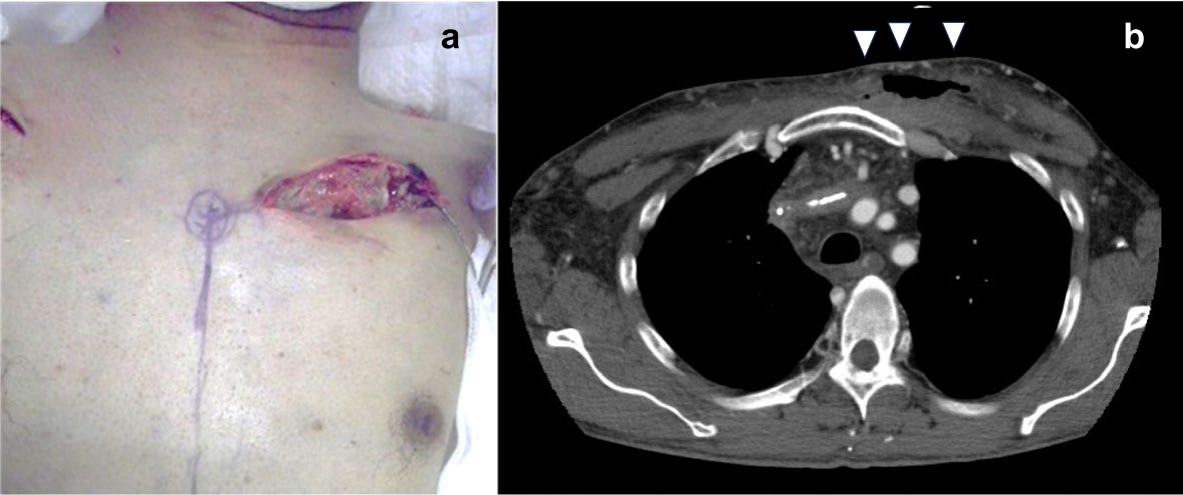
Open wound in the left anterior thorax (**a**) and chest CT (**b**). **a** The generator was removed and the RV lead remained. A subcutaneous pocket was formed in the midline and (**b**) extended to the anterior surface of the sternum (arrowheads)

A tissue culture of skin fistula was positive for methicillin-sensitive *Staphylococcus aureus* (MSSA). An attempt was made to remove the infected device and leads via a transvenous approach using both an excimer laser and a mechanical sheath. The generator and two RA leads were extracted but the two RV leads couldn’t be extracted (Fig. 1b). He was therefore referred to our department for surgical removal. The open wound in the left anterior thorax had a pocket, the inner edge of which reached the anterior surface of the sternum (Fig. 2). A surgical approach with a sternotomy was found to associated with high risk of DSWI.

Contrast-enhanced computed tomography (CT) showed that the superior vena cava (SVC) was completely occluded from the brachiocephalic vein to the SVC-RA junction by the four leads that had been implanted. The remaining two RV leads were detached to the middle of the SVC in the previous procedure. Based on these two findings, it was determined that extracorporeal circulation with RA-inferior vena cava (IVC) cannulation via the femoral vein would provide a bloodless field in the right heart system and lead extraction could be performed. Therefore, we decided to use an endoscopic minimal invasive cardiac surgery (MICS) approach to leads extraction.

**Fig. 3:**
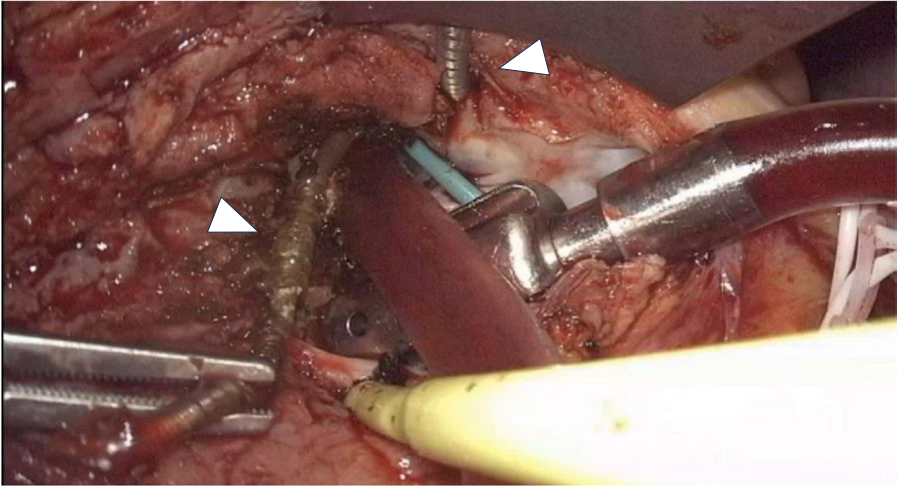
Intraoperative view. The right atrial wall was inflamed and thickened, and the upper half of the right atrium was occupied by an organizing thrombus. There were two right ventricular leads through the tricuspid annulus (arrowheads)

A camera port was created on the right fourth intercostal mid-axillary line, a 3-cm main incision was made on the right fourth intercostal anterior axillary line, and a 5-mm left-hand port was made in the third intercostal space. Extracorporeal circulation was established by cannulation through the right femoral artery with an 18Fr. PCKC (SENKO MEDICAL INSTRUMENT Mfg.Co., LTD) and vein with a 25Fr. HLS cannula (Getinge). The wall of the right atrium was thickened. The RA was opened without cross-clamping after IVC snaring. The upper half of the RA lumen was occupied by an organizing thrombus and SVC was occluded. As there was backflow from the RV, repeated short circulatory arrests at 28℃ were used. Two ventricular leads were dissected and removed but one was heavily adherent to the RV and cut near the level of the annulus (Fig. 3). After completing the endocardial manipulation, a small incision was made in the left sixth intercostal space and an epicardial lead was placed in the left ventricular lateral wall. The generator was placed in the upper abdomen (Fig. 1c).

**Fig. 4:**
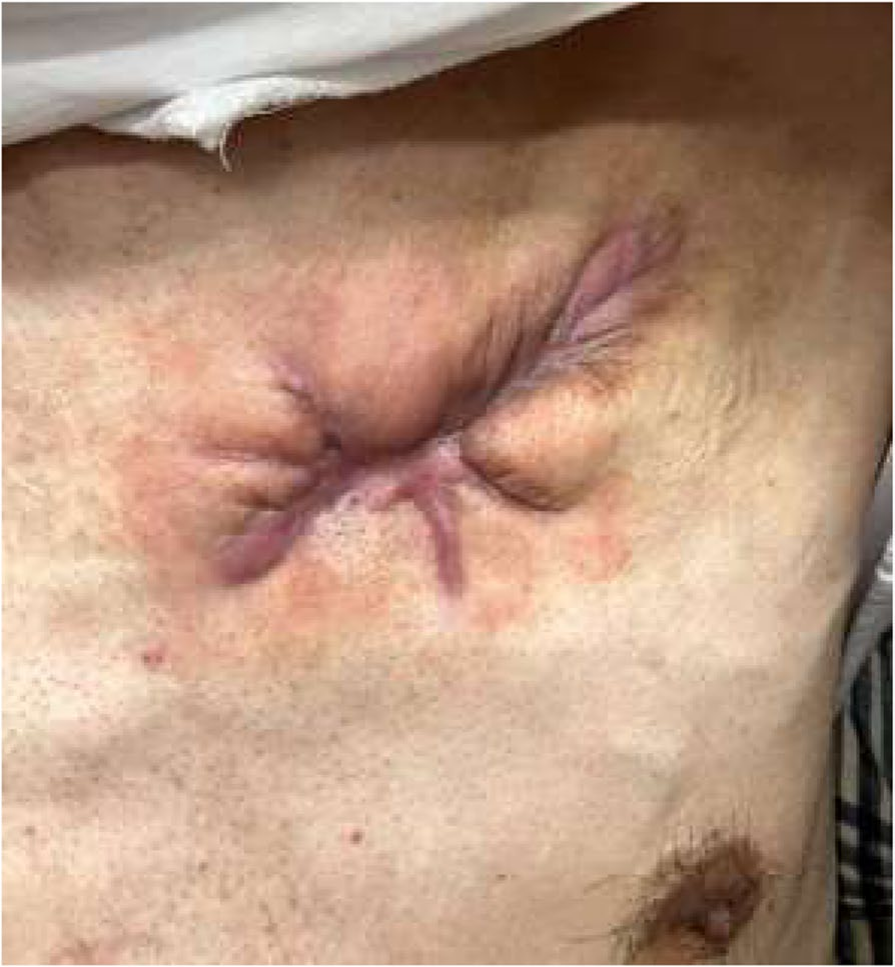
Healed wound. No surgical site infection was observed after device removal. Following granulation occurred, segmental skin grafting was performed and healing was achieved

The clinical course was good and there was no surgical site infection. Tricuspid regurgitation was trace and didn’t change after surgery. The open wound was healed with radical debridement and segmental skin grafting after granulation (Fig. 4).

## Discussion and conclusions

The risk of a CIED infection has been reported to be 0.5%-1% and 1%-5% for a first-time procedure or replacement and upgrade, respectively. The associated mortality is not low (5%-17%) [1]. Treatment of a CIED infection requires the appropriate antibiotics and device removal. There are two main methods for CIED lead removal: TLE and surgical. TLE is a less invasive approach than surgery; however, the incidence of major complications is 1.8%-2.3% and catastrophic complications that require emergency surgical or endovascular intervention occur in 0.3%-0.8%. Moreover, the complication site is the SVC in 56% of cases [2, 3]. Lead-related risk factors for major complications include a greater number of extracted leads and a longer duration of lead implantation [3].

Surgical extraction is indicated in TLE failure cases, in patients at high-risk for complications, and in patients with complex lesions, large vegetations (> 2.5 cm), or the need for an epicardial lead implantation. The procedure generally requires a sternotomy and cardiopulmonary bypass, and therefore is more invasive than a TLE, and there is a high risk for DSWIs. Mediastinitis is associated with a mortality rate of 9.7%-29% and the long-term prognosis is poor [3–6]. The MICS approach may be less invasive than a surgical approach with a sternotomy and is more difficult to perform on peripheral veins than the SVC.

In our case, the wound was opened and the pocket was reached above the sternum. Surgical extraction was required due to TLE failure, but it was important to avoid making a median sternotomy. Fortunately, the remaining two RV leads were dissected from the SVC during the prior TLE procedure. Therefore, we could perform an endoscopic MICS approach via a small right thoracotomy. The MICS approach had three advantages. First, the RA wall was thickened by inflammation, therefore a lateral approach was appropriate for viewing the RV through the tricuspid valve. Second, the surgical incision was distant from the infected site in the anterior thorax bilaterally and a surgical site infection didn’t occur. Third, an injury for resternotomy could be avoided.

A hybrid TLE and MICS approach may be preferable in cases in which the TLE procedure alone is difficult or in patients at high risk for a DSWI.

## Abbreviations

CIEDs: Cardiac implantable electronic devices
CT: Computed tomography
DSWI: Deep sternal wound infection
IVC: Inferior vena cave
MICS: Minimal invasive cardiac surgery
MSSA: Methicillin-sensitive *Staphylococcus aureus*
PM: Pacemaker
RA: Right atrium
RV: Right ventricle
SVC: Superior vena cave
TLE: Transvenous lead extraction
